# Lifestyle and Chronic Constipation in Medical Students

**DOI:** 10.1155/2021/4752614

**Published:** 2021-01-04

**Authors:** Mehmet Aykut Yildirim, Murat Cakir, Mehmet Bicer, Mustafa Senturk, Harun Yonar, Merve Nur Gur, Zeliha Nur Akiner, Ayse Guldiken, H. Kaan Karagul, Bugra Ceri

**Affiliations:** ^1^Necmettin Erbakan University, Meram Medical Faculty, Department of General Surgery, Konya/, Turkey; ^2^Selçuk University, Faculty of Veterinary Medicine Department of Biostatistics, Turkey

## Abstract

**Aim:**

Constipation is one of the most common complaints of the digestive system indicated with an increase in defecation frequency, difficulty in defecation, and hard and strained defecation. Environmental, personal, and genetic factors may be affecting constipation although the affecting factors have not yet been thoroughly explained. The aim of this study was to investigate constipation frequency and lifestyles in medical students.

**Method:**

The population was selected among medical students for the study, which was planned as a survey study. Demographic data of all the participants and the factors suggested to affect constipation were questioned and analyzed.

**Results:**

The study covered a total of 425 medical students. Among the students reporting constipation, 2.86% were in their first year of medical school, while 7.53% were in the third year and 9.09% were in the sixth year. The rate of students reporting constipation and familial history was statistically significant. While regular eating habits were reported in the first and third years, this rate was much lower in the sixth year group working at clinical departments. The results of our study did not reveal any significant relationship between daily intake of water and constipation. There was, however, a significant relationship between stress and constipation.

**Conclusion:**

The results of our study showed that medical education did not curb constipation frequency. We believe that stress is significant in constipation. The data we collected indicate that regular eating habits and excess liquid intake are not as effective as suggested in the treatment of constipation.

## 1. Introduction

As one of the most common complaints of the digestive system, constipation is in fact not a disease, but a symptom [1]. Patients complaining about constipation may express different problems. Some patients mention a reduction in defecation frequency, while some others refer to difficulty in defecation with hard to pass stools.

Constipation is usually defined as defecation frequency less than three times a week [2]. Yet 60% of patients complaining about constipation defecate daily. Such patients also complain about long-lasting defecation, straining taking up most of the time, and a lingering feeling of discomfort [2]. These complaints indicate functional constipation [3]. An international study committee determined the diagnostic criteria for functional constipation (Rome IV) [3].

The prevalence of constipation varies among societies, age groups, and the characteristics of individuals questioned. With an estimated prevalence of 15% (12-19%) in the general population [4], it is more common in women and individuals over 60 years of age. The frequency is affected by the chronic diseases and lifestyles of the individuals. Factors affecting constipation have not yet been explained thoroughly. Numerous environmental, personal, and genetic factors may affect constipation.

Therefore, the aim of this study was to investigate constipation frequency and lifestyles in medical students (healthy young individuals).

## 2. Method

This study was planned as a voluntary survey. The questionnaire included 15 questions to investigate the participants' defecation conditions and factors affecting constipation. The study population was selected among the first-, third-, and sixth-year undergraduates studying at Necmettin Erbakan University Meram Medical School. A minimum participation of 80% was aimed for the students of all these years. These students were selected considering that the first-year students had not taken medical education yet and better reflected the general society. Third-year students, on the other hand, had completed their basic medical education but not started clinical training yet. Having completed their medical training, sixth-year students were working as doctors. These groups reflected three different stages in medical training. The study was conducted between January 1, 2019, and June 1, 2019, after obtaining the approval of the local board of ethics.

The demographic data and the factors considered to affect constipation were questioned and analyzed. The bowel habits of the participants were also investigated. The screening parameters included age, sex, history of abdominal surgery, medication used, presence of constipation, medication used for constipation, familial history of constipation and/or intestinal cancer, regular exercise, eating habits, amount of water intake, sleeping patterns, stressful life, watching weight, and seeing a doctor for constipation. Constipation diagnosis was evaluated according to ROME IV criteria. Patients with the form of Irritable Bowel Syndrome concurring with constipation were not evaluated to have constipation.

Exclusion criteria included individuals with inflammatory bowel disease, chronic metabolic, endocrinal and neurological disorders, and a history of surgical treatment for constipation.

### 2.1. Statistical Analysis

Categorical data collected by the questionnaire were given through crosstabs including percentage and frequency figures. Chi-square test was used to compare and determine any differences in frequency among the groups within the scope of crosstab analyses. Exact or Monte Carlo methods were used for calculations depending on data count and table type in Chi-square assessments. Significance level was determined as 5% in the statistical evaluations, and SPSS version 22 software was used for analyses.

## 3. Results

The study covered 425 participants including 140 first, 186 third, and 99 sixth-year medical students, respectively. 47 of the first-year students were male and 93 were female and their mean age was 18.36. 88(47.3%) of the third-year students were male and 98(52.7%) were female and their mean age was 21.56. 55(55.6%) of the sixth-year students were male and 44(44.4%) were female and their mean age was 23.82. There was a statistically significant relationship between the years and the sex variable (*p* < 0.05). While 33.6% of the participants in the first year were male, this figure increased to 55.6% in the sixth year indicating an unsimilar distribution of sexes in each year.

Among the participants reporting constipation, 2.86% were in the first year of medical school while 7.53% were in their third year and 9.09% were in their sixth year. 11 (7.9%) first year, 26 (14.0%) third year, and 6 (6.1%) sixth-year students were on chronic medication. Among the students using medication for constipation, 1.43% were first year, 1.08% were third year, and 2.02% were sixth-year students.

10% of the first-year students, 20.4% of third-year students, and 19.2% of sixth-year students reported constipation in their family members, while 7.1% of the first-year student, 18.4% of third-year students, and 21.1% of sixth-year students also had constipation themselves. 20.4% of the third-year students reported a familial history of constipation, while 18.4% were constipated as well. The results of our study revealed a significant relationship between the years and the familial history of constipation variable (*p* < 0.05). This indicated a statistically significant difference between these rates. The percentages of students presented to a doctor for constipation were 7.9%, 3.8%, and 6.1% in the first-, third-, and sixth-year students, respectively.

Based on the eating habits of the participants, it was seen that first- and third-year students had more regular eating habits while this rate was lower in the sixth year students working at clinical departments. Yet the difference was not statistically significant. The majority of the students consumed 1-2 liters of water daily. There was no difference among the groups. There was no relationship between water intake and constipation either. The rate of students who exercised regularly was about 20%. Third- and sixth-year students exercised more regularly compared to first-year students. While 40.7% of the first years watched their weights, this figure went up to 57.0% and 68.7% in third- and sixth-year students, respectively. It was seen that as the students spent more time at medical school, they watched their weights more closely but this was not statistically significant.

More than 50% of the students reported irregular sleeping patterns and stress. 69.3% of the first-year students reported that they had stress and 4.1% of these students were constipated. 86.9% of the third-year students reported that they had stress, and 11% of them were constipated. 84.6% of the sixth-year students reported that they had stress, and 9.3% of them were constipated. The constipation rate of those experiencing stress was higher in all years, and this was statistically significant (*p* < 0.05).

Previous abdominal surgery rate was below 10% in all groups (*p* < 0.371).

All collected data were summarized in Table 1.

## 4. Discussion

Normal bowel order is not uniform. Numerous factors and personal characteristics may affect bowel habits. Extended colonic transition time or defecation dysfunction is observed in patients with chronic constipation. These two main conditions are responsible for chronic constipation. Reduced intestinal motility results in constipation. Constipation is usually defined as the slow transition of intestinal content from the proximal to the distal. Such slowing is generally observed in the colon. It is more distinct in the distal part of the colon. This condition is called slow transit constipation [2, 3]. Although this slowing can be associated with an ample number of causes, most cases are grouped under the idiopathic type with no identifiable cause. All these conditions are referred to as chronic constipation. Based on ROME IV criteria, constipation cannot be diagnosed only according to defecation count. The patient should be evaluated based on defecation way and difficulty, feeling of relief, anorectal obstruction, feeling of blockage, and hand-aided defecation rates. Description of constipation by the patients may be different from its description in the field of medicine. Colorectal malignity, neurologic, metabolic, and endocrinal disorders and chronic medication administration play a significant role in secondary chronic constipation [4]. The approximate prevalence of constipation in the general population is 15% [1, 2]. Female sex and advanced age are the two important responsible factors. The population of our study included young, healthy, and educated individuals. The frequency of constipation in our study was 6.35%. The results of our study revealed that constipation frequency was 2.86%, 7.53%, and 9.09% in the first-, third-, and sixth-year medical students, respectively. Age and the inclusion of healthy and active individuals constitute the cause for lower frequency in our study compared to literature. We observed some increase in the prevalence with age. The most significant disadvantage of our study group was that they spent more time on the toilet.

Objective and subjective factors are effective in functional constipation diagnosis [5]. The most common symptom in patients examined for chronic constipation is extreme straining. Hard feces, abdominal discomfort, and swelling follow this symptom. Everyone expresses constipation in a different way. It is difficult to standardize this definition. We tried to form a more standard group including young individuals as our cases. We evaluated the individuals with constipation complaint according to ROME IV criteria.

The most important treatment approach for chronic constipation is the clarification of etiological factors. The treatment of chronic constipation incorporates patient training, behavioral modification, dietary changes, and laxative treatment. Severe and persistent slow-transit constipation is rare and may require surgical treatment [6]. Defecation training is important for patients with chronic constipation. Patients should regulate their lifestyles. The gastrocolic reflex after eating, particularly after breakfast, facilitates defecation [7, 8]. Defecation after eating is recommended to capitalize on this reflex. Individuals need to develop regular bowel habits [6]. Regular eating, adequate fiber, and fluid intake are effective in preventing constipation [7]. Chronic diseases and medication for them trigger constipation. In such cases, it would be right to review the medication used. Abuse of laxatives negatively affects bowel habits. Regular eating is important in ameliorating bowel habits. Our results showed that our subjects had regular eating habits. The rates of those exercising regularly, however, were not higher than 20%. We detected an inadequate fluid intake since most cases consumed fluid less than 2 liters. Nevertheless, we did not ascertain a relationship between regular eating habits and insufficient liquid intake with constipation. We also observed a high rate of compulsory deviations in lifestyles. A great majority of our cases reported stressful lifestyles. Our results, accordingly, showed a close relationship between stressful lifestyle and constipation. Only a few of our cases were on constipation medication.

## 5. Conclusion

Our results revealed that medical training did not reduce constipation frequency. We believe that stress is a significant factor in constipation. Our data also showed that regular eating and excess liquid intake were not as effective as suggested in constipation treatment.

## Figures and Tables

**Table 1:** Demographic data.

Mean age ± SD (min-max)		Years			
		First year (*n*: 140)	Third year (*n*:186)	Sixth year (*n*: 99)	*p*
		18.37 ± 0.703 (17-21)	21.56 ± 1.909 (20-43)	23.82 ± 0.850 (22-27)	
Sex (*n* (%))	Male	47 (33.6%)	88 (47.3%)	55 (55.6%)	0.002^∗^
	Female	93 (66.4%)	98 (52.7%)	44 (44.4%)	
History of abdominal surgery (*n* (%))	Yes	7 (5%)	17 (9.1%)	7 (7.1%)	0.371
	No	133 (95%)	169 (90.9%)	92 (92.9%)	
Are you on any medication? (*n* (%))	Yes	11 (7.9%)	26 (14.0%)	6 (6.1%)	0.057
	No	129 (92.1%)	160 (86.0%)	93 (93.9%)	
Are you constipated? (*n* (%))	Yes	4 (2.9%)	14 (7.5%)	9 (9.1%)	0.103
	No	136 (97.1%)	172 (92.5%)	90 (90.9%)	
Are you on medication for constipation? (*n* (%))	Yes	2 (1.4%)	1 (0.5%)	2 (2.0%)	0.533
	No	138 (98.6%)	185 (99.5%)	97 (98.0%)	
Does your family have constipation? (*n* (%))	Yes	14 (10.0%)	38 (20.4%)	19 (19.2%)	0.038^∗^
	No	126 (90.0%)	148 (79.6%)	80 (80.8%)	
Does your family have intestinal cancer? (*n* (%))	Yes	5 (3.60%)	17 (9.10%)	7 (7.10%)	0.134
	No	135 (96.40%)	169 (90.90%)	92 (92.90%)	
Regular exercise (*n* (%))	Yes	18 (12.9%)	39 (21.0%)	18 (18.2%)	0.167
	No	122 (87.1%)	147 (79.0%)	81 (82.4%)	
Eating habits (*n* (%))	Regular	85 (60.7%)	121 (65.1%)	53 (53.5%)	0.161
	Irregular	55 (39.3%)	65 (34.9%)	46 (46.5%)	
Amount of water intake (liter (L))	Less than 1 L	43 (30.7%)	40 (21.5%)	20 (20.2%)	0.195
	1-2 L	71 (50.7%)	106 (57.0%)	60 (60.6%)	
	2-3 L	18 (12.9%)	34 (18.3%)	17 (17.2%)	
	More than 3 L	8 (5.7%)	6 (3.2%)	2 (2.0%)	
Sleeping patterns (*n* (%))	Regular	66 (47.1%)	78 (41.9%)	39 (39.4%)	0.463
	Irregular	74 (52.9%)	108 (58.1%)	60 (60.60%)	
Stress (*n* (%))	Yes	97 (69.3%)	155 (83.3%)	86 (86.9%)	0.001^∗^
	No	43 (30.7%)	31 (16.7%)	13 (13.1%)	
Weight watching (*n* (%))	Yes	57 (40.7%)	106 (57.0%)	68 (68.7%)	0.143
	No	83 (59.3%)	80 (43.0%)	31 (31.3%)	
Have you seen a doctor for constipation? (*n* (%))	Yes	11 (7.9%)	7 (3.8%)	6 (6.1%)	0.285
	No	129 (92.1%)	179 (96.2%)	93 (93.9%)	

Mean ± SD, ^∗^Chi-square Test, ^∗^(*p* < 0.05).

## Data Availability

The data used to support the findings of this study are available from the corresponding author upon request.
